# Isolated Pulmonary Artery Sling Diagnosed in Adulthood

**DOI:** 10.1002/ccr3.71195

**Published:** 2025-10-06

**Authors:** Emine Afsin, Zeliha Coşgun, Rukiye Öztürk

**Affiliations:** ^1^ Department of Chest Diseases Abant Izzet Baysal University Bolu Turkey; ^2^ Department of Radiology Bolu Abant Izzet Baysal University Bolu Turkey

**Keywords:** adulthood, congenital vascular ring anomaly, isolated, pulmonary artery sling

## Abstract

Although pulmonary artery sling (PAS) is typically a congenital vascular ring anomaly detected in early childhood, its isolated form can also be diagnosed in adulthood. While respiratory symptoms are commonly expected, chest pain should also raise suspicion for PAS.

## 1 Case

**FIGURE 1:**
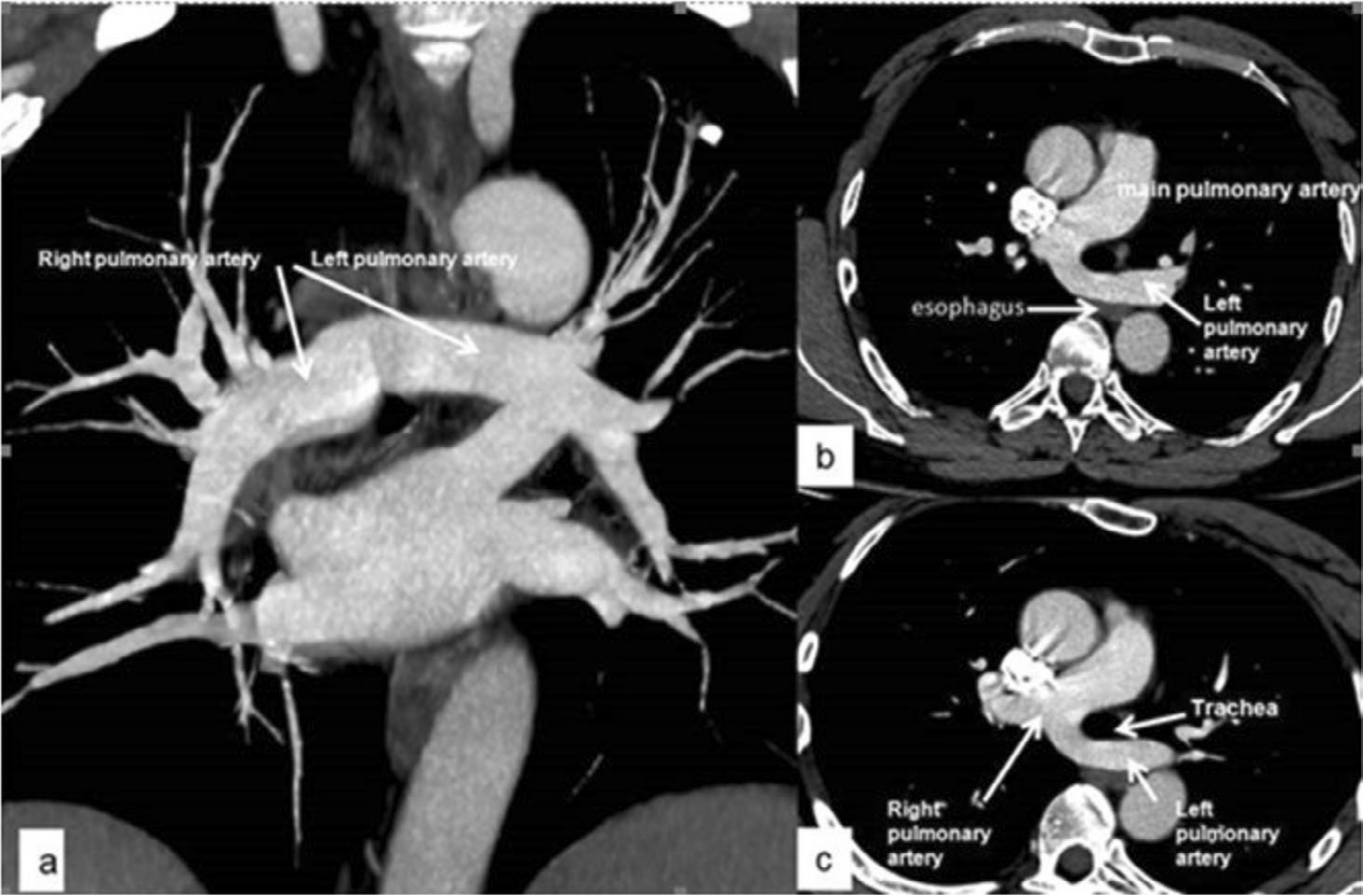
Contrast‐enhanced thoracic computed tomographic scan in the mediastinal window, coronal (a) and axial (b, c) planes, demonstrates that the left main pulmonary artery originates from the right pulmonary artery and courses posterior to the trachea and anterior to the esophagus before reaching the left lung hilum, consistent with a pulmonary artery sling.

A 48‐year‐old male patient presented to the outpatient clinic with a 3‐month history of dull, non‐pleuritic chest pain on the left side. The pain was unrelated to exertion, body position, or eating. The patient had no other active respiratory or gastrointestinal complaints but reported frequent acute bronchitis during childhood and a diagnosis of tuberculosis 12 years ago. His respiratory system examination was normal, and no abnormalities were found in biochemistry and hemogram values, including cardiac enzymes. The electrocardiogram showed sinus rhythm without ischemic changes. As no abnormalities were observed on the chest X‐ray, thoracic computed tomographic angiography was performed. It revealed that the left pulmonary artery originated from the right pulmonary artery and coursed posterior to the trachea and anterior to the esophagus before reaching the left lung hilum (Figure 1), suggesting a pulmonary artery sling (PAS). When echocardiography was performed, no cardiac anomaly was detected. Echocardiography revealed no cardiac abnormalities. Following consultation with cardiovascular surgery, a conservative, non‐surgical follow‐up plan was adopted.

A PAS describes an abnormal left pulmonary artery passing between the trachea and esophagus just above the level of the carina in most cases. An anastomotic vessel arising from the right pulmonary artery and connecting the primitive pulmonary circulations becomes the abnormal left pulmonary artery. The estimated incidence of PAS is 59 per million live births, with approximately 90% of cases diagnosed in the first year of life [1].

Patients with PAS can be classified into two main groups based on accompanying lesions and age at presentation. The first and more common type is associated with congenital heart defects and anomalies of the tracheobronchial tree, causing respiratory distress and feeding difficulties in early childhood. These cases have high morbidity and mortality rates. The less common isolated form may remain asymptomatic or present with dysphagia and/or respiratory symptoms in adulthood. In our case, the diagnosis may have been delayed until adulthood due to the isolated nature of the anomaly.

While all pediatric patients diagnosed with PAS should undergo surgical correction, surgery is rarely performed in adults. According to the literature, only a few adult cases with severe symptoms due to tracheal compression have been treated surgically [2]. In our case, surgery was not considered, as the patient did not exhibit significant symptoms.

Adult cases are rare, with few reported worldwide. Our case is notable because it is an isolated case presenting with chest pain in adulthood. Chest pain is rare and may result from compression of adjacent organs by the vascular ring. In our patient, since no other pathology was identified to explain the chest pain, it was considered that PAS might be the underlying cause.

In conclusion, congenital anomalies can also be detected in adulthood and should be considered in the differential diagnosis of chest pain.

## Author Contributions

**Emine Afsin:** conceptualization, data curation, methodology, supervision, writing – original draft, writing – review and editing. **Zeliha Coşgun:** conceptualization, data curation, visualization. **Rukiye Öztürk:** conceptualization, methodology.

## Ethics Statement

Written informed consent was obtained from the patient for publication of this case report and any accompanying images. A copy of the written consent is available for review by the Editor of this journal and provides consent to publish as‐NA.

## Consent

The patients provided written informed consent to publication of this report.

## Data Availability

The datasets used and/or analyzed during the current study are available from the corresponding author on reasonable request.

## References

[1] J. M. Yu , C. P. Liao , S. Ge , et al., “The Prevalence and Clinical Impact of Pulmonary Artery Sling on School‐Aged Children: A Large‐Scale Screening Study,” Pediatric Pulmonology 43 (2008): 656–661. 18484662 10.1002/ppul.20823

[2] F. Huang , Q. Q. Lai , H. Wu , and X. T. Ke , “A Left Pulmonary Artery Sling in an Asymptomatic Adult Patient, A Case Report and Review of Literature,” Heart Surgery Forum 24 (2021): E278–E281. 33798043 10.1532/hsf.3637

